# Excellent supercapacitance performance of 3-D mesoporous carbon with large pores from FDU-12 prepared using a microwave method†

**DOI:** 10.1039/c8ra01281d

**Published:** 2018-05-09

**Authors:** Wang Soo Cha, Siddulu Naidu Talapaneni, Devaraju M. Kempaiah, Stalin Joseph, Kripal Singh Lakhi, Abdullah M. Al-Enizi, Dae-Hwan Park, Ajayan Vinu

**Affiliations:** Global Innovative Center for Advanced Nanomaterials (GICAN), Faculty of Engineering and Built Environment, The University of Newcastle (UON) Callaghan NSW 2308 Australia SidduluNaidu.Talapaneni@newcastle.edu.au Ajayan.Vinu@newcastle.edu.au +61-2-4921-8669; Future Industries Institute (FII), Division of Information Technology Energy and Environment (DivITEE), University of South Australia Adelaide SA 5095 Australia; Department of Science, King Saud University Riyadh Saudi Arabia

## Abstract

Highly ordered and three-dimensional (3-D) mesoporous carbon materials were prepared through a nano-hard templating approach using FDU-12 silica with tunable pore sizes as a template, which was synthesized *via* a microwave-assisted method. Powder XRD and microscopic techniques such as HR-TEM, HR-SEM, and N_2_ adsorption–desorption techniques were employed to characterize the structure and textural properties of the prepared mesoporous carbon samples. The characterization results reveal that all the mesoporous carbon samples show a 3-D porous mesostructure with tunable pore diameters (5.7 to 9.4 nm) and a large specific surface area in the range from 451 to 1251 m^2^ g^−1^. The supercapacitive behavior of the cubic structured mesoporous carbons was determined using cyclic voltammetry, electrochemical impedance and charge–discharge measurements. The cubic mesoporous carbon materials exhibit a superior capacitive performance with a high specific capacitance value of 315.3 F g^−1^ at the current density of 1 A g^−1^, which is much higher than that of hexagonally-ordered mesoporous carbon with large pore diameters, activated carbon, and carbon nanotubes. The materials also show excellent cyclic stability and extremely low resistance. The superior specific capacitance of these materials is attributed to the combination of excellent surface properties such as large specific surface area, large pore volume and uniform pore diameter, spherical morphology, and a 3-D porous system with cage-type pores.

## Abstract 2

Highly ordered and three-dimensional (3-D) mesoporous carbon materials were prepared through a nano-hard templating approach using FDU-12 silica with tunable pore sizes as a template, which was synthesized *via* a microwave-assisted method.

## Graphical abstract

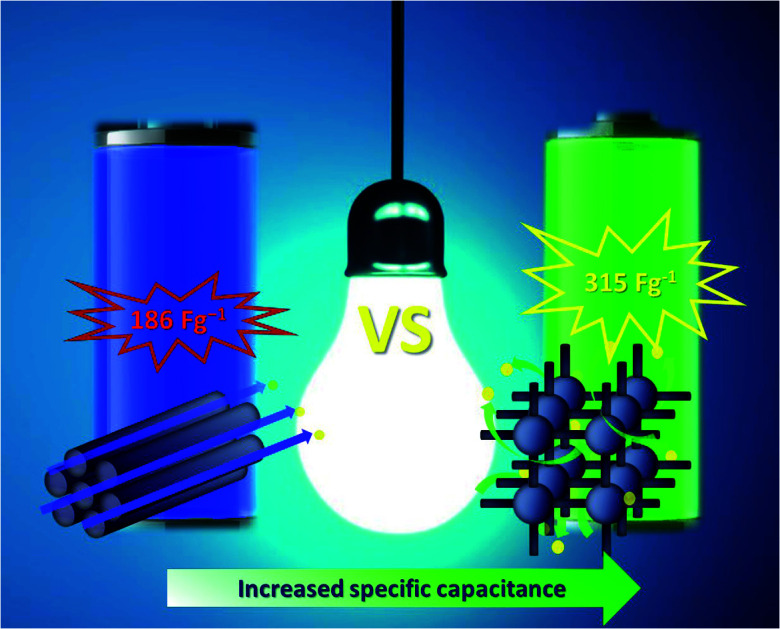

## Introduction

The significant increase in the use of energy resources due to rapid urbanization and population growth has become an increasing concern in the recent years. Moreover, the indiscriminate use of fossil fuels has led to considerable depletion of energy resources, climate change and damage to the environment due to pollution. These problems have prompted a number of researchers to find sustainable and eco-friendly solutions to meet future energy demands.^1–6^ Electrochemical energy storage devices, including supercapacitors and batteries, are a possible solution, as they are promising, stable and robust clean energy technologies and have the capability to deliver and store energy quickly.^6–12^

In recent years, supercapacitors have received huge attention, and have steadily been developed as an alternative to conventional energy storage devices. Supercapacitors have shown much better reversibility, remarkable capacitance, fast charging and discharging at high power densities and high stability when compared to other energy devices such as secondary batteries and dielectric capacitors.^3,5,12–18^ However, at high current densities, supercapacitors yield a low power density and specific capacitance, and the efficiency of the lifecycle also decreases in the long run.^19,20^ These drawbacks can be overcome by employing nanostructured electrode materials. Therefore, much attention has been given to improving the specific capacitance and efficiency of supercapacitors by fabricating electrode materials with high surface area, high conductivity, and uniform morphology to facilitate the rapid diffusion of electrons.^21,22^

To date, a number of materials such as conducting polymers, mesoporous carbons, and metal oxides have been extensively employed as electrodes in supercapacitors, owing to their unique structural characteristics, including excellent conductivity, high surface area, chemical stability and unique electronic properties.^1,22,23^ Recently, hexagonally-ordered 2-dimensional mesoporous carbon CMK-3 (2-D CMK-3) was used as an electrode material due to its high surface area and pore volume, as well as tunable pore diameter.^1,22^ Dattatraya *et al.* reported that the pore diameter and specific surface area of an electrode material are the key parameters that dictate the final specific capacitance of a device.^1^ It was demonstrated that CMK-3 with the largest pore diameter and the highest specific surface area, which was prepared by using SBA-15 with a large pore diameter, exhibits the highest specific capacitance.^1^ It was assumed that a large capacitance could be achieved by creating active materials with improved textural parameters and unique morphologies, as these characteristics support easy electrolyte diffusion within the porous channels of the mesoporous carbon electrodes.

To control the morphology of mesoporous carbon electrodes, the morphology of the hard template, mainly mesoporous silica, should first be controlled. Microwave radiation-assisted synthesis approaches have been generally used for controlling the morphology of the templates, as they have the ability to provide uniform and volumetric heating throughout the samples. These special features of microwave-assisted synthesis can enhance the reaction rate and promote the formation of uniform nucleation centers, which will eventually offer uniform and controlled morphology.^24–26^ The microwave approach also significantly reduces the synthesis time of mesoporous materials from 72 hours to less than 2 hours. This significant reduction in the synthesis time will help to reduce the costs associated with the preparation of the materials and pave the way for quick commercialization.

The structure of electrode materials plays a significant role in controlling the final textural parameters and the performance of the electrodes in supercapacitors.^27^ It has been proven that carbide-derived porous carbon with a 3-D cubic structure is a promising electrode material for supercapacitor applications, as it offers much better ion transport performance due to its highly interconnected porous network.^27,28^ However, reports on the supercapacitance performance of mesoporous carbon with a controlled morphology, highly ordered and 3-D structure are quite limited. In this work, we report on the synthesis of a highly ordered 3-D mesoporous carbon material for the first time using a FDU-12 silica template, which was prepared by a microwave-assisted method, as a high-performance electrode material for supercapacitors. The textural parameters, including the pore diameters of the mesoporous carbon, have been fine-tuned by using mesoporous silica templates with a 3-D structure prepared at various synthesis temperatures. These materials showed a long lifecycle with an exceptionally high specific capacitance of 315.3 F g^−1^ at the current density of 1 A g^−1^, which is much higher than that of CMK-3 with a 2-D structure.^1^

## Experimental section

### Preparation of FDU-12 by microwave irradiation

In a typical synthesis, 2 g of Pluronic F127, 2 g of mesitylene, and 3 g of KCl were dissolved in 120 mL of 2 M HCl solution. The mixture was stirred for 4 h at 25 °C. After that, 8.3 g of tetraethyl orthosilicate was slowly added to the mixed solution, and the resultant mixture was kept at 35 °C for 24 h. The mixture was then transferred to a microwave oven and heated at 200 °C for a period of 2 h. A series of samples were prepared using the same procedure but treated at different temperatures (100, 130, and 150 °C). Each sample was then filtered and washed with deionized water (DI), followed by drying at 100 °C for 6 h and calcination at 540 °C for 12 h. The prepared mesoporous silica samples were named FDU-12-M-*T*, where *T* denotes the temperature used for the synthesis of FDU-12 and M denotes the microwave.

### Synthesis of mesoporous carbon with three-dimensional and cage-type pores

To prepare the cubic-structured mesoporous carbon, 1 g of pre-calcined FDU-12-M-*T* was thoroughly mixed with a solution containing 0.9 g of sucrose, 0.1 g of H_2_SO_4_, and 3.5 g of DI water. The mixture was polymerized at 100 °C for 6 h, and then at 160 °C for another 6 h. In order to achieve the complete filling of the pores with the carbon source, a second filling was carried out by adding the mixture to 3.5 g of DI water containing 0.75 g of sucrose and 0.084 g of sulfuric acid, and the resulting mixture was kept at 100 °C and then 160 °C, for 6 h each. After crushing, the composite was carbonized in a nitrogen atmosphere for 6 h at 900 °C, and the FDU-12 silica template was removed by HF treatment. In order to remove the HF, the composite was washed repeatedly with ethanol and dried for 6 h. The final mesoporous carbon samples were denoted as MCF-M-*T* (*T* = 100, 130, 150 and 200 °C).

### Electrode preparation

For the electrochemical measurements, electrode materials were prepared by mixing 90 wt% of MCF-M-*T* materials and 10 wt% of polyvinylidene fluoride (PVDF) in isopropanol. The slurry was coated onto nickel foam, and the nickel foam was pressed and then dried at 100 °C in the vacuum oven overnight.

## Results and discussion

### Formation of mesoporous silica and mesoporous carbon

**Scheme 1:**
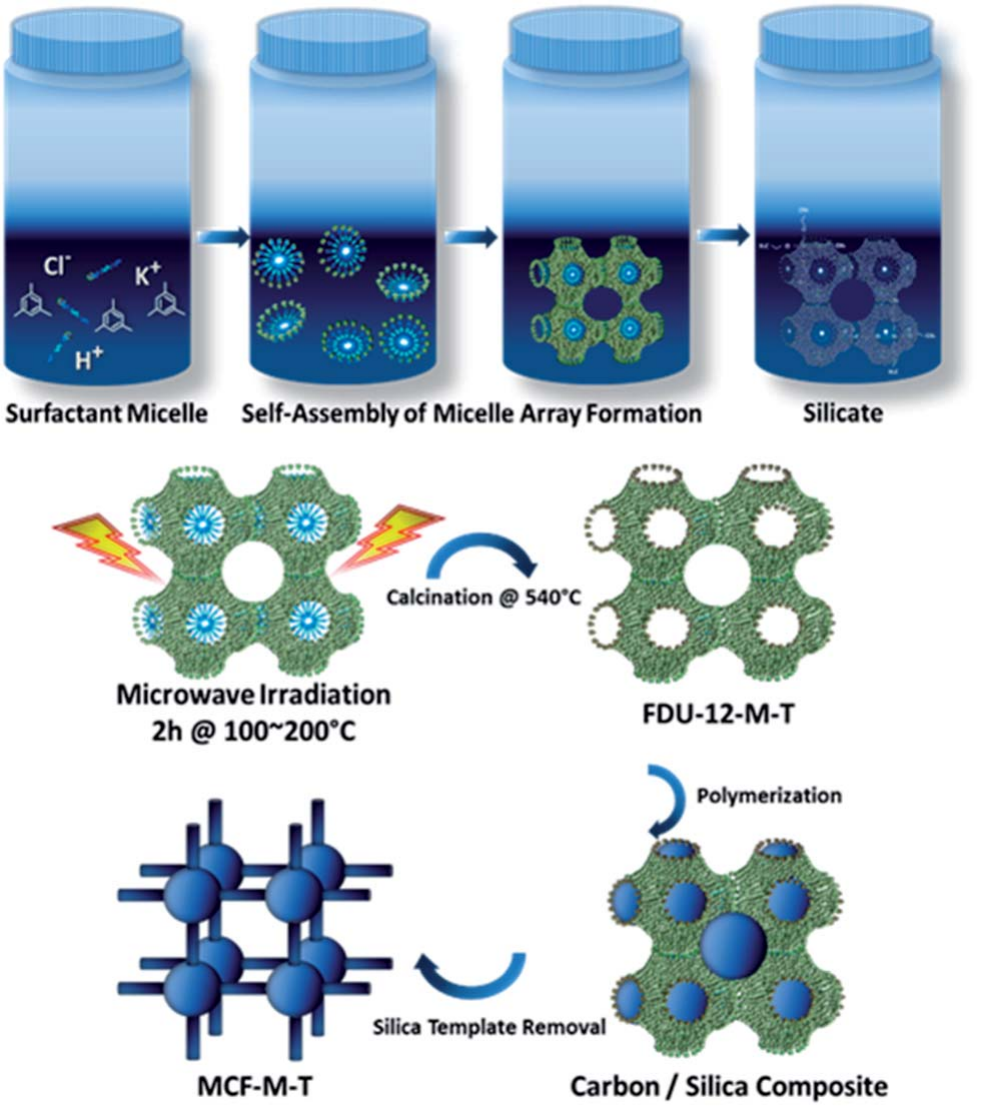
Schematic illustration presenting the preparation of cubic-structured MCF-M-*T*.

The preparation of the mesoporous carbon materials with 3-D porous structures was carried out using a nano-hard templating approach, wherein samples of mesoporous silica (FDU-12-M-*T*), with a 3-D cage-type porous structure and tunable pore sizes prepared *via* a microwave approach were used as the templates. Scheme 1 shows the synthetic route adopted for the preparation of mesoporous silica, FDU-12-M-*T*, using a microwave approach and its subsequent conversion to highly ordered mesoporous carbon with a 3-D cage-type structure. First, F127 non-ionic triblock copolymer, containing both ethylene oxide and propylene oxide (EO106PO70EO106) played a crucial role in directing the formation of the inorganic mesopores. The micelles were self-assembled through the packing of spherical micelles of surfactant, and were formed with cage-type meso-phase in the acidic solution. Microwave irradiation was applied to reduce the reaction time for obtaining highly ordered mesoporous silica with a controlled morphology and 3-D structure. The microwave irradiation approach not only significantly reduces the synthesis time from 72 h to 2 h but also helps in obtaining a uniform spherical morphology through uniform and volumetric heating. The fabrication process of the mesoporous carbon includes the infiltration of sucrose into the mesopore channels of the FDU-12-M-*T* silica template, followed by the polymerization of the carbon precursors with sulfuric acid as a catalyst and carbonization treatment at 900 °C under a nitrogen environment, and finally the removal of the silica templates with dilute aqueous HF solution. This hard-templating approach allows the structure and morphology of the mesoporous FDU-12-M-*T* templates to be copied onto the MCF-M-*T* materials.

### Analysis of structure and morphology

**Fig. 1:**
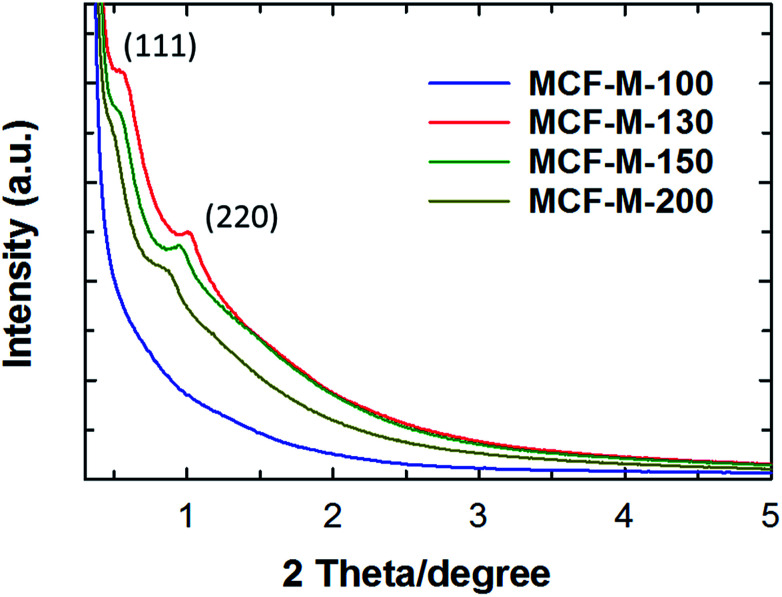
Powder XRD patterns of MCF-M-*T* synthesized by microwave irradiation of FDU-12 followed by carbonization.

The powder XRD patterns of the MCF-M-*T* samples prepared using FDU-12-M-*T* with different pore sizes are shown in Fig. 1. Two diffraction peaks are observed at lower angles from 0.3 to 5 degrees (2*θ*) for the samples MCF-M-130, MCF-M-150 and MCF-M-200, corresponding to the crystal planes of (1 1 1) and (2 2 0) and a large lattice constant. These results reveal the formation of a face-centered cubic structure in the *Fm*3*m* space group, which is similar to the parent silica template (Fig. S1†).^29^ The intensity of the (1 1 1) reflection increases with decreasing the synthesis temperature of the template, except for the MCF-M-100 sample. This is due to the large mesopores that are formed by the incomplete filling of large cages in the templates prepared at a high temperature. This creates a weak-intensity peak due to the interference of the diffracted X-rays between the empty pores and the carbon walls. No diffraction peak was observed for the MCF-M-100 sample, indicating low crystallinity or an amorphous structure. This could be due to the small pore diameter of the template, which gives a smaller wall thickness. This does not fully support the perfect cross-linking of the carbon precursors with the neighboring pore channels, which is necessary for the complete replication process, leading to the collapse of structure during the carbonization. Among the MCF-M-*T* samples, the MCF-M-130, MCF-M-150 and MCF-M-200 samples showed sharp peaks at lower angles, confirming better crystallinity in terms of the pore structural order. This is quite a unique result, as it is very difficult to maintain a good structural order for mesoporous carbon materials prepared from hydrothermally-synthesized templates at temperatures higher than 150 °C.^30^

**Fig. 2:**
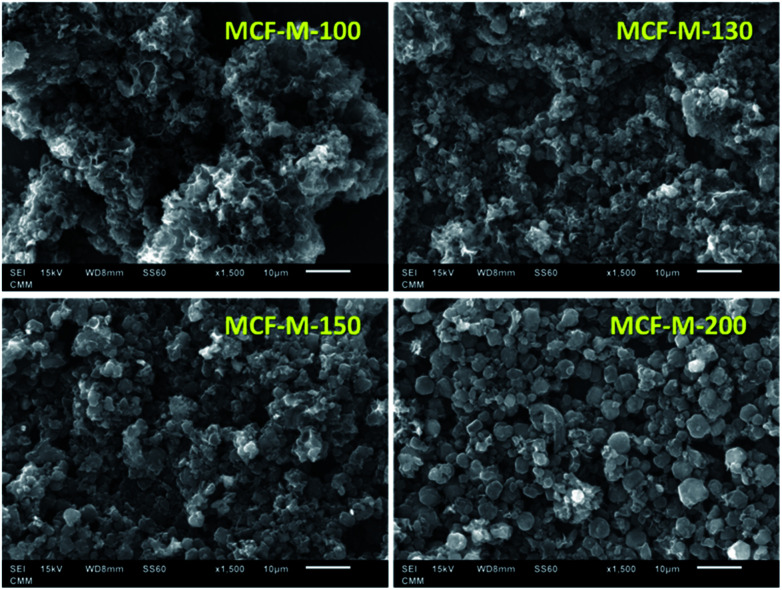
SEM images of MCF-M-*T* samples synthesized by microwave irradiation followed by carbonization at 900 °C for 6 h.

The morphology of the samples was analyzed by HR-SEM, as shown in Fig. 2. From the SEM images, it can be seen that the MCF-M-*T* materials show spherical morphology with a porous structure and the particles are connected together to form intra-particle pores. It should be noted that the morphology of the prepared carbon materials is slightly different from that of the mesoporous silica templates, as the spherical particles of the carbon materials are partially open and linked together (Fig. S2†). It is also observed that some of the samples exhibit sphere-like microscale particles that are formed inside the macro-particles, which are wide open, and the size of these particles is calculated to be *ca.* 2 to 3 μm in diameter (Fig. S3†). From the SEM images, it is also clear that the materials are highly macroporous with different morphologies, which changes by altering the synthesis temperature of mesoporous silica. The retention of slightly distorted spherical morphology confirms that the replication process was almost successful.

**Fig. 3:**
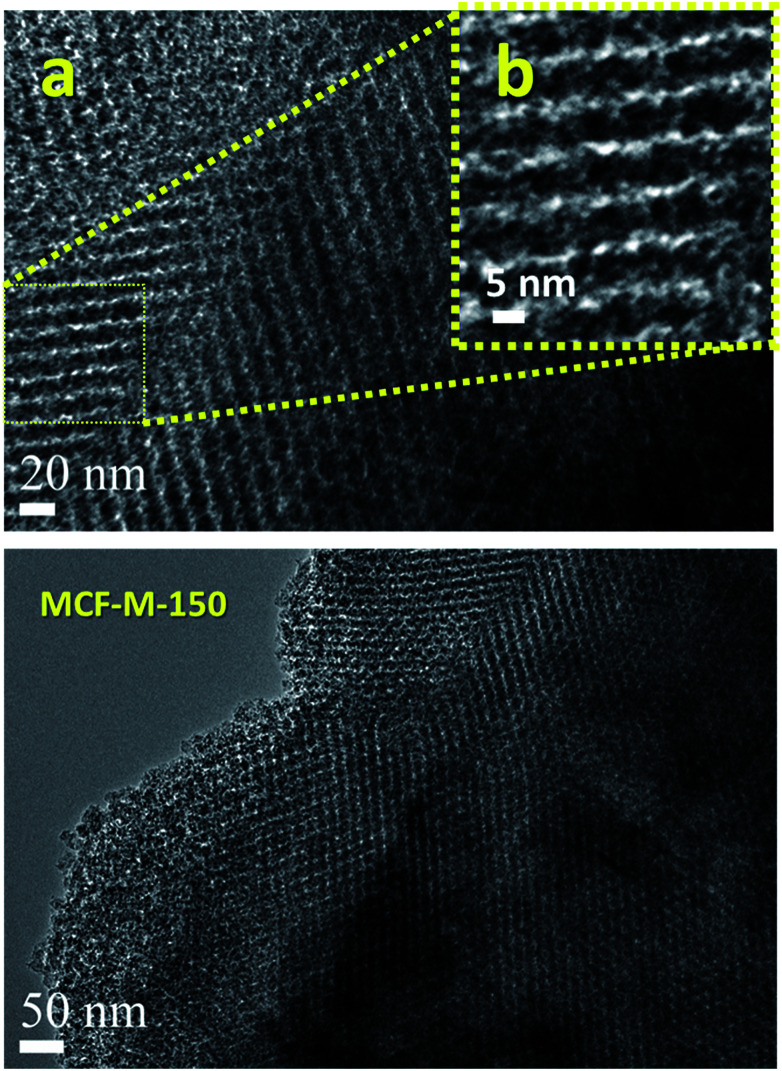
HR-TEM images of the MCF-M-150 samples exhibiting porous morphologies ((b) is an enlarged segment from (a)).

Fig. 3 shows the HR-TEM images of the MCF-M-150 sample. It is interesting to note that the HR-TEM images of the sample exhibit 3-D interconnected mesopores throughout the large domains. The pore structure of the sample is almost similar to that of the FDU-12 silica templates (Fig. S4 and S5†). This indicates that the morphology of the sample does not affect the pore structure of the materials. MCF-M-150 has a well-ordered cubic structure with *Fm*3*m* symmetry, as well as a highly ordered, linear array of mesochannels without any intergrowths. The pore channels with inter-connected and highly organized pores in a 3-D network can be clearly observed using high-magnification HR-TEM. The pores are organized in regular intervals with a high surface roughness, as can be seen in the high-resolution image of the sample (Fig. 3 inset). The lattice parameter derived from this image is very similar to that obtained from the XRD data, confirming the perfect replication of the 3-D mesoporous structure into the corresponding mesoporous carbon molecular sieves. This kind of unique porous structure can provide more electrochemically-active sites, which will be helpful for enhancing the supercapacitive performance with a fast ion diffusion and randomly directional ion movement during the electrochemical reaction.

**Fig. 4:**
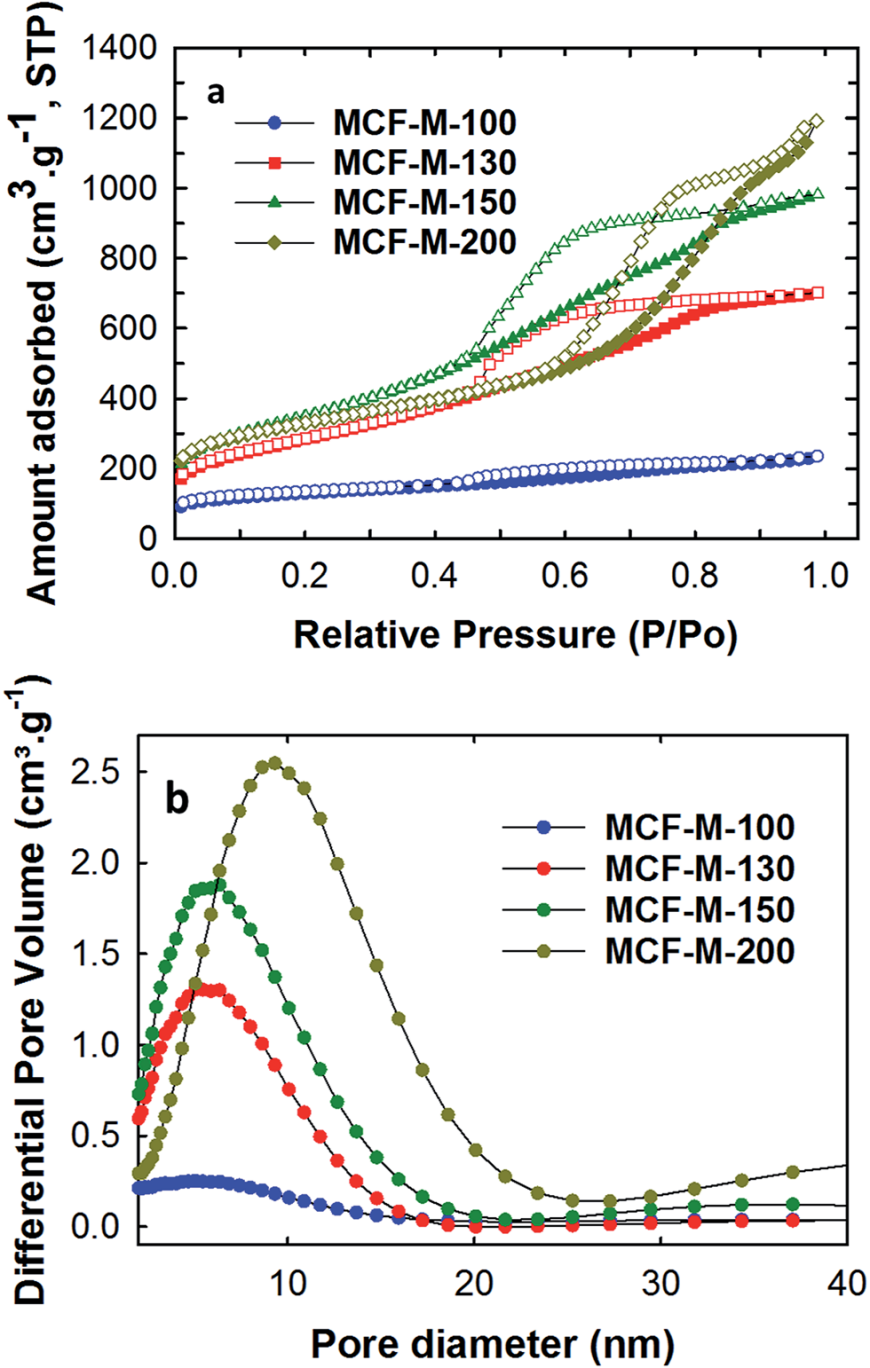
(a) Nitrogen absorption–desorption isotherms; (b) pore diameter distribution of the MCF-M-*T* samples.

Nitrogen adsorption–desorption analysis was carried out for all of the samples to obtain the surface parameters, including the pore sizes, the specific surface areas, and the specific pore volumes of the MCF-M-*T* samples. Fig. 4 shows the nitrogen adsorption–desorption isotherms of the MCF-M-*T* samples. Type IV isotherms are observed for all the samples and they display a H2 hysteresis loop, which is characteristic of 3-D mesoporous materials with a cage-type porous structure. The quantity of the nitrogen adsorbed increases as the synthesis temperature of the silica templates is increased from 100 to 150 °C but decreases afterwards. It is worthy of note that the size of the hysteresis loop increases as the pore diameter of the template is increased. Among the samples studied, MCF-M-200 shows the largest hysteresis loop, confirming the presence of large cage-type pores. This is further established by the pore size distribution of MCF-M-*T*, which shows that the mesopores are highly regular and uniform. The pores become slightly broadened as the synthesis temperature of the FDU-12 silica template is increased.

The increase of the synthesis temperature of the templates has a significant effect on the final textural parameters, including the pore diameter of the mesoporous carbon and silica samples, as shown in Table 1 and Fig. S6.† It was observed that the BET surface area of the as-obtained samples increases from 451.3 to 1251.5 m^2^ g^−1^ with an increase of the synthesis temperature of the FDU-12 silica templates from 100 °C to 150 °C. The BET surface area then decreases slightly to 1164.8 m^2^ g^−1^ as the synthesis temperature is further increased to 200 °C. However, the pore volume and the pore diameters gradually increase when the synthesis temperature of the mesoporous silica is increased. This is confirmed by the shift of the capillary condensation step of the isotherms towards higher relative pressure, whereas the multi-layered adsorption step is shifted upwards for MCF-M-200. The pore volume and pore diameter of the samples are in the range of 0.365–1.844 cm^3^ g^−1^ and 5.7–9.4 nm respectively. It is also interesting to note that the MCF-M-100 sample exhibited the lowest surface area and pore volume of 451.3 m^2^ g^−1^ and 0.365 cm^3^ g^−1^, respectively. This reveals that the lowest pore diameter of the template prepared at the lowest synthesis temperature is not suitable for obtaining ordered mesoporous carbon with excellent textural parameters. Among the samples studied, MCF-M-150 exhibits the highest specific surface area, while the MCF-M-200 sample exhibits the largest specific pore volume, which originates from the large pore diameter.

**Table 1:** Textural properties of MCF-M-*T* samples

Material	*A*_BET_ (m^2^ g^−1^)	*V*_p_ (cm^3^ g^−1^)	*d*_p,BJH_ (nm)
MCF-M-100	451.3	0.365	—
MCF-M-130	1024.2	1.085	5.7
MCF-M-150	1251.5	1.519	6.2
MCF-M-200	1164.8	1.844	9.4

### Electrochemical studies

**Fig. 5:**
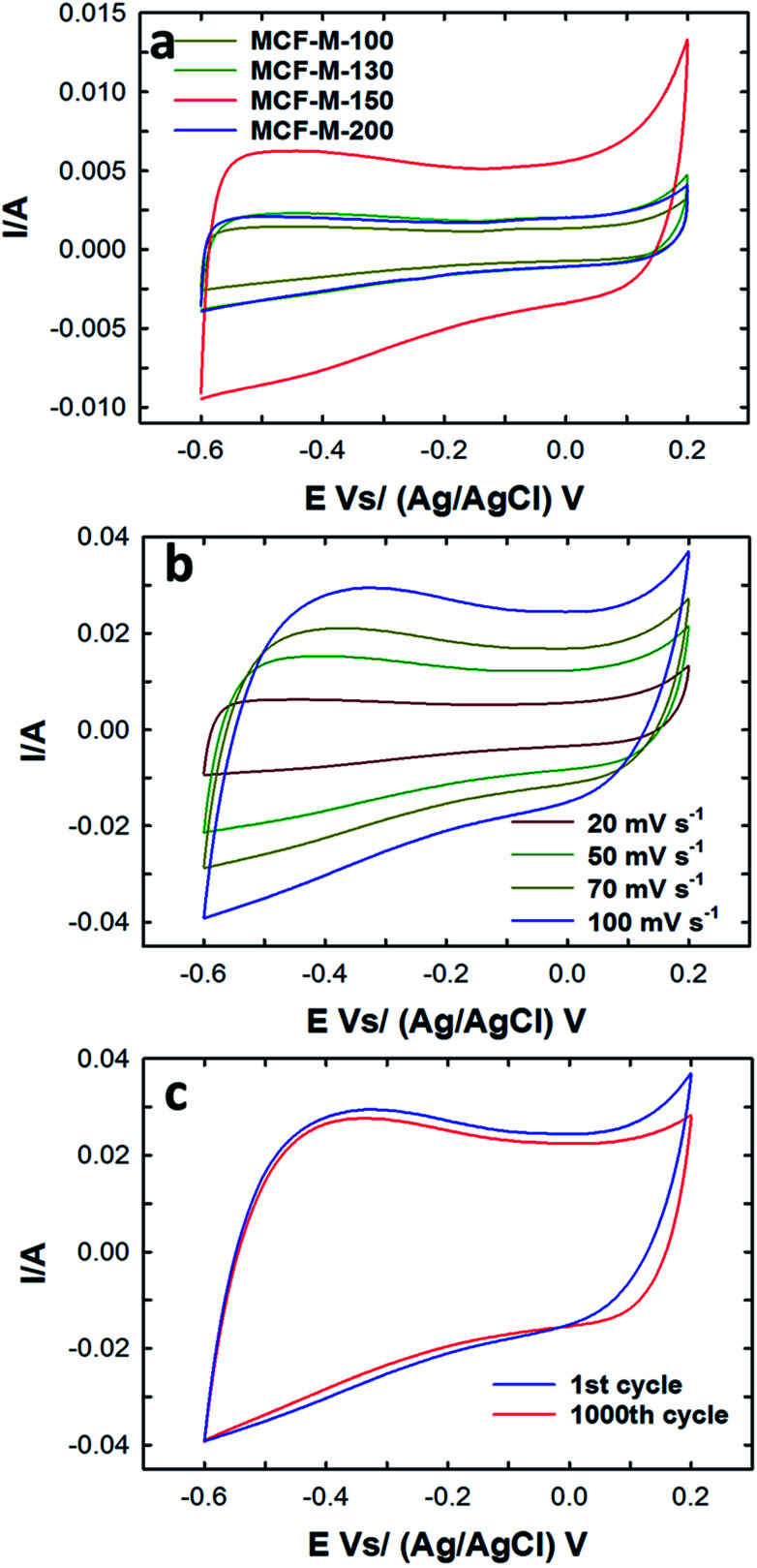
(a) CV profiles of the MCF-M-*T* samples at a constant scan rate of 20 mV s^−1^; (b) CV profiles of the MCF-M-150 at a different scan rate; (c) CV cyclability of the MCF-M-150 at a constant scan rate of 100 mV s^−1^.

The electrical double layer capacitance was measured by using a three-electrode cell assembly made from the mesoporous carbon materials prepared in this work. First, cyclic voltammetry measurements of these cells were obtained in order to understand the effect of different textural parameters on the electrochemical performance of MCF-M-*T*-based electrodes (Fig. S7†). Fig. 5a represents the typical CV curves for the MCF-M-*T* electrodes with different pore diameters and specific surface areas at a constant scan rate of 20 mV s^−1^ in 2 M KOH electrolyte using a Pt wire and Ag/AgCl as a counter and reference electrode, respectively, in the potential range between −0.6 and 0.2 V. As can be seen in Fig. 5, the CV curves of MCF-M-130, 150 and 200 are almost rectangular in shape, revealing the good capacitive behavior of the carbon materials. It is also believed that rapid ion transport inside the nanopore channels by short ion diffusion distances and multi-directional ion movement in the cubic-structured mesoporous carbon materials with spherical morphology supports the formation of a rectangular shape in all the CV curves. In other words, fast and reversible adsorption of the ions of the electrolyte onto the electrochemically-active sites of the mesoporous carbon electrodes with cubic porous structures significantly enhances the capability to store more energy at the interface between the surface of the electrode and electrolyte.

Among the samples studied, the area under the curve of the MCF-M-150-based electrode is much larger than that of the other samples investigated in this work. This reveals that MCF-M-150 has a much stronger capacitive behavior and faster ion transport than the other mesoporous carbon electrodes. The increase in the capacitance of MCF-M-150 can be ascribed to the fact that MCF-M-150 has a much better structural order and higher specific surface area than the other mesoporous carbon electrodes, which improves access to the electrochemical active sites on the surface of the walls of these electrodes. On the other hand, the low capacitance of MCF-M-100 is due to the fact that the electrolyte accessibility is limited in the material with the disordered porous structure and poor textural parameters, including low specific surface area and narrow pore diameter, which offer only a limited number of electrochemically active sites. At different scanning rates, none of the curves exhibit peaks between −0.6 and 0.2 V, showing that the charging and discharging electrodes exhibit a pseudo-constant rate during the voltammetric cycle. The shape of the curve at the scanning rate of 100 mV s^−1^ is still retained and only a little reduction in the specific capacitance is observed, revealing that the sample maintains a high capacitive behavior with an excellent rate capability even at a high sweep rate, and the equivalent series resistance (ESR) is quite small. This is extremely important for obtaining a higher power density. It is believed that the 3-D cubic structure of the carbon electrode supports easy ion transport within the mesochannels with a short diffusion distance, which ultimately maintains a good capacitive behavior even at a higher scan rate. The long-term electrochemical stability of the MCF-M-150 sample was also examined by measuring a large number of cycles in the CV measurements, and the data are presented in Fig. 5c. The MCF-M-150-based electrode shows a very stable charge–discharge process even after 1000 cycles, revealing that the material has a robust wall structure which is quite stable, allowing the material be used for repeated cycles. This property is extremely important for practical electrochemical energy storage applications.

Taking electrochemical measurements of galvanostatic charging and discharging is a more reliable and accurate method for calculating the specific capacitance compared to cyclic voltammetry. The specific capacitance of the samples at different current densities was measured using charge–discharge profiles. The capacitance of the MCF-M-*T* samples was also calculated at different current densities using the following equation:

1 *C*_S_ = (*IT*_D_/*E*_X_*W*)

where *I* is current (A), *T*_D_ is the discharging time in seconds, *E*_X_ is the range of voltage in V, and *W* is the weight of the active materials in g.

**Fig. 6:**
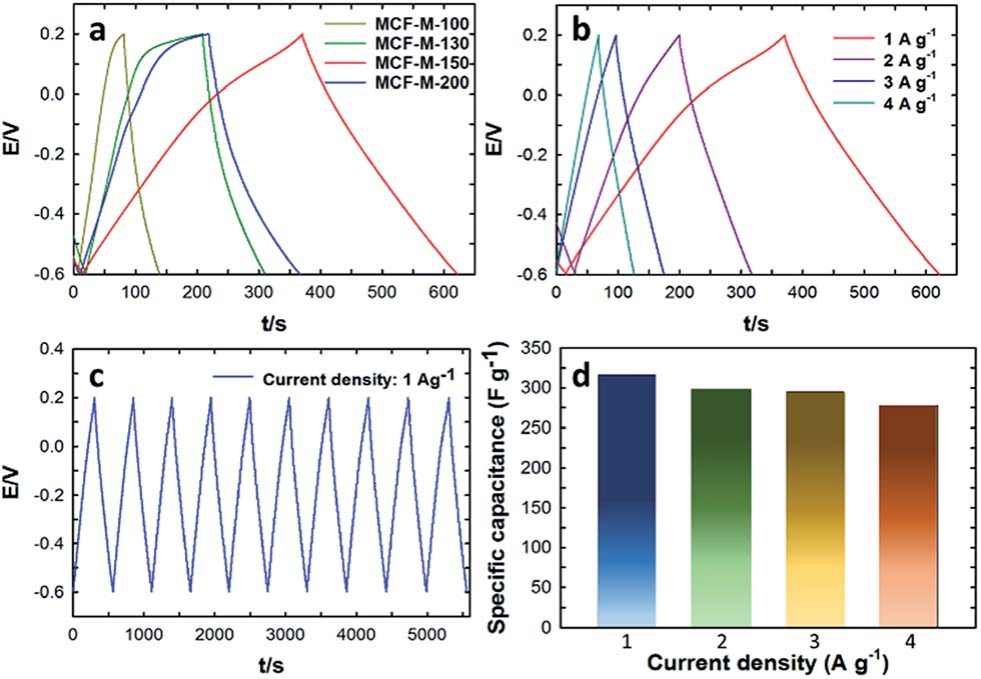
(a) Charge–discharge profiles of MCF-M-*T* samples at a current density of 1 A g^−1^; (b) charge–discharge profiles of the MCF-M-150 sample at different current densities; (c) cyclic performance of MCF-M-150; (d) calculated specific capacitance at different current densities of MCF-M-150.

Fig. 6a–c shows the galvanostatic charge–discharge results of the MCF-M-*T* electrode materials examined using the chronopotentiometry technique at different current densities in the potential range of −0.6 to 0.2 V. All materials show a triangular shape as well as a low drop of the ESR with a constant slope in the charge–discharge curve. These results indicate that the electrode materials have a good cyclability rate with a small ESR and a high power density. A rapid change and very low drop at the beginning of the charge–discharge curve, which is associated with the ESR as commonly seen from the capacitive behavior of EDLC, was also observed. As can be seen in Fig. 6a, the specific capacitance was increased linearly with increasing specific surface area of the mesoporous MCF-M-*T* electrodes. A maximum specific capacitance of 315.3 F g^−1^ at the current density of 1 A g^−1^ was achieved for the MCF-M-150 sample, while those of MCF-M-100, MCF-M-130 and MCF-M-200 were 75.1, 130.2 and 185.1 F g^−1^, respectively. From these results, it is clear that the higher specific capacitance of MCF-M-150 is mainly due to its larger specific pore volume and specific surface area as compared to those of the other materials used in this study. It is surmised that these superior textural parameters combined with a 3-D mesoporous structure reduce the diffusion resistance of the solvated electrolyte ions and further enhance the access to a greater number of electrochemically active sites, resulting in higher specific capacitance.

The charge–discharge profiles of the MCF-M-150 sample at different current densities from 1 A g^−1^ to 4.0 A g^−1^ are also shown in Fig. 6b. The charge–discharge curves of the MCF-M-150 sample exhibit symmetric shapes without an obvious IR drop, and show excellent reversibility and a high Coulombic efficiency and specific capacitance. This result indicates that the mesoporous carbon material MCF-M-150 has an excellent rate capability, and the 3-D porous cubic structure of the MCF-M-150 is highly favorable for electrolyte ion transport and charge transfer reactions. The combination of the large pore diameter, pore volume and high specific surface area of the MCF-M-150 material allows the solvated electrolyte ions to access the active sites easily and helps to avoid diffusional limitations, but assists the easy formation of an electrical double layer between the electrode and electrolyte interface. This significantly enhances the contact between the electrode and electrolyte ions, resulting in the highest specific capacitance. The highest specific capacitance value of 315.3 F g^−1^ was obtained at a current density of 1 A g^−1^ with stable capacitance retention (Fig. 6d). This is the highest ever specific capacitance value reported for mesoporous carbon-based electrodes (Table 1S†). It should be noted that the specific capacitance decreases from 315.3 to 277.6 F g^−1^ as the current density increases from 1 to 4 A g^−1^, respectively (Fig. 6d). To be precise, the specific capacitance is reduced by 5.5% as the current density is increased from 1 to 2 A g^−1^, while only the specific capacitance is decreased by 1.0% when the current density is increased from 2 to 3 A g^−1^. This also shows that MCF-M-150 achieved a rapid ion diffusion and excellent electrochemical utilization due to its high specific surface area.

**Fig. 7:**
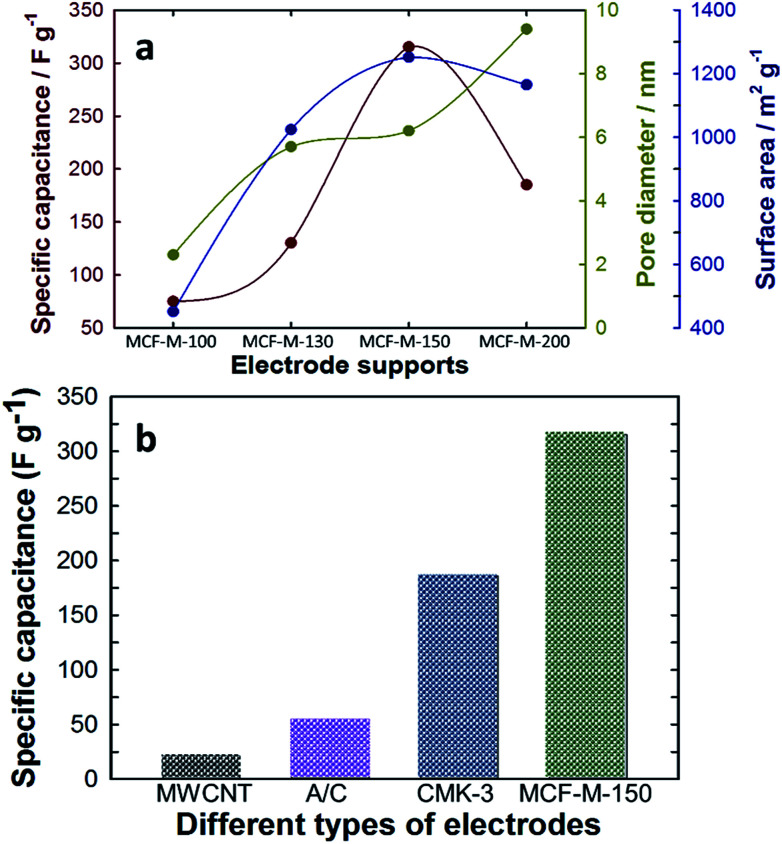
(a) Variation of specific capacitance with textural parameters of the MCF-M-*T* samples; (b) comparison of specific capacitance with MWCNT, A/C, CMK-3-150 and MCF-M-150.

The relation between the specific capacitance of the MCF-M-*T* samples with the pore diameter and surface area is summarized in Fig. 7a. The results clearly indicate the influence of the textural properties of each MCF-M-*T* sample on the specific capacitance. The pore diameter and surface area are mainly responsible for the substantial increase in the specific capacitance of MCF-M-150. Furthermore, the specific capacitance of MCF-M-150 was also compared with other carbon electrodes based on MWCNT (22 F g^−1^), activated carbon (AC) (55 F g^−1^), and mesoporous carbon CMK-3-150 (186 F g^−1^) materials (Fig. 7b and Table 1S†). It was found that the MCF-M-150-based electrode shows remarkable performance over the other carbon-based electrodes studied in this work. In particular, the MCF-M-150-based electrode with a 3-D structure achieved almost 2 times higher specific capacitance than the CMK-3-150-based electrode with a 2-D structure. This superior enhancement in the electrochemical performance of the MCF-M-150-based electrode is mainly attributed to the 3-D cubic porous structure, which facilitates effective transportation and rapid circulation of ions in all directions through the interconnected mesopore channels, while the 2-D structured mesoporous carbon-based electrodes show limited and unidirectional transport of ions.^21^

To further understand the performance of these materials in a two-electrode system, we fabricated 2032 standard coin cells using MCF-M-150 as an active material. As a result, the materials exhibited a high specific capacitance of 251.7 F g^−1^ at a current density of 0.15 A g^−1^ (Fig. S8†). The observed results are interesting and reveal the ability of these electrode materials to perform in any cell configuration.

**Fig. 8:**
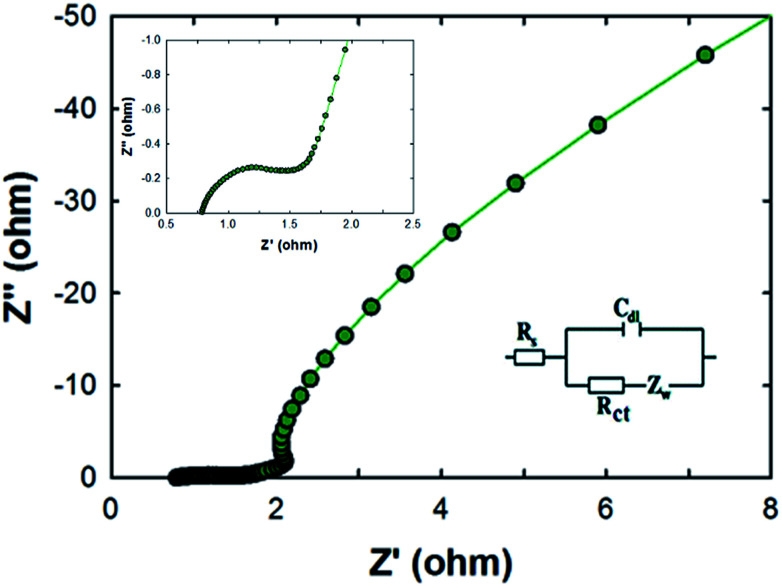
Nyquist plots of the MCF-M-150 sample from 10 mHz to 1 MHz. The inset is an expanded profile in the region of high frequencies and an equivalent circuit modelling.

Electrochemical impedance spectroscopy (EIS) measurements were carried out for the MCF-M-150 electrode in the frequency range of 1 MHz to 10 mHz with an amplitude of 0.005 V. As can be seen in Fig. 8, two distinct circles were observed, showing both a semicircle at the high-frequency range and an inclined line at the lower-frequency range. The inset shows the Randle circuit diagram exhibiting the total resistance, ionic resistance, intrinsic resistance of active material and the resistance of the current collector. The semicircle in the high-frequency range results is mainly due to the surface interaction of the mesoporous carbon material corresponding to the faradic charge-transfer resistance (which is equivalent to *R*). It is also noted that the MCF-M-150 electrode material has a very small *R*_s_ of 0.77 Ω, which indicates low impedance at the electrolyte–electrode interface. Additionally, the Warburg angle was proven to be higher than 45°. These results strongly suggest that the MCF-M-150 electrode, with a cubic structure and uniform distribution of mesopores in the active material, could be an ideal electrode material for supercapacitor applications.^31,32^

## Conclusion

In summary, we have demonstrated for the first time the synthesis of highly ordered 3-D cage-type mesoporous carbon materials, prepared from FDU-12 silica with tunable pore diameters as hard templates, which were prepared using an ultra-fast and energy efficient microwave-assisted approach. We have also demonstrated their application as electrode materials for supercapacitors. The use of silica templates prepared using a microwave-assisted approach reduced the time and overall energy consumption in the synthesis of mesoporous carbon materials as compared to using a conventional hydrothermal route. The carbon materials prepared in this work show excellent surface parameters such as large pore diameter, high specific surface area, and large pore volume, which are critical parameters for enhancing the specific capacitance of supercapacitors. It was found that the electrode materials with the highest surface area and largest pore volume showed the highest supercapacitance of 315.3 F g^−1^ at the current density of 1 A g^−1^, which is much higher than that of electrode materials prepared from 2-D CMK-3 carbon materials, MWCNT, and activated carbons. Furthermore, the materials prepared in this work also exhibit long-term cyclability and very low resistance. This superlative electrochemical performance is attributed to the combined effect of excellent textural parameters and 3-D porous structures, which facilitate better electrolyte ion diffusion than their 2-D counterparts or disordered activated carbons and MWCNT. It is surmised that this simple microwave approach could be applied for the fabrication of a series of mesoporous carbon-based materials from different templates, paving the way towards designing low-cost and high-performance electrochemical supercapacitors.

## Conflicts of interest

There are no conflicts to declare.

## Supplementary Material

RA-008-C8RA01281D-s001
