# Supplementary material for: Excellent supercapacitance performance of 3-D mesoporous carbon with large pores from FDU-12 prepared using a microwave method
Source: RSC Adv. 2018 May 9;8(31):17017–24. doi: 10.1039/c8ra01281d (PMC9080457; doi:10.1039/c8ra01281d)
Supplement: RA-008-C8RA01281D-s001 [file RA-008-C8RA01281D-s001.pdf]

Electronic Supplementary Material (ESI) for RSC Advances.
This journal is © The Royal Society of Chemistry 2018

Supplementary Information

Excellent supercapacitance performance of 3D mesoporous carbon with large pores from FDU-12 prepared by microwave method

Wangsoo Cha[a], Siddulu Naidu Talapaneni[a], Devaraju M. Kempaiah[b], Stalin Joseph[a], Kripal Singh Lakhi[a], Abdullah M. Al-Enizi[c], Dae-Hwan Park[a], Ajayan Vinu[a]**

[a] Global Innovative Center for Advanced Nanomaterials (GICAN), Faculty of Natural Built Environment and Engineering, School of Engineering, The University of Newcastle, Callaghan, NSW 2308, Australia.

[b] Future Industries Institute, Division of Information Technology Energy and Environment, University of South Australia, Adelaide, SA 5095, Australia

[c] Department of Science, King Saud University, Riyadh, Saudi Arabia

*To whom the correspondence should be addressed

Email: SidduluNaidu.Talapaneni@newcastle.edu.au

Ajayan.Vinu@newcastle.edu.au

Ph: +61-2-4921-8669

Characterization

The morphology of the samples was determined by HR-SEM (JEOL JSM 6610) operating at an acceleration voltage of 15kV and the working distance of 8 mm. HR-TEM images of the samples are obtained from JEOL JEM-2000EX2 operating at an acceleration voltage of 200 kV. The powder X-ray diffraction (XRD) pattern measurements were recorded on a Rigaku diffractometer equipped with Cu kα radiation (λ=0.154 nm), The diffraction peaks were recorded by selecting 2θ range from 0.3° to 5° with a step size of 0.01 per second. The nitrogen adsorption and desorption measurements were carried out on a Micromeritics ASAP2420. Before the analysis, all the samples were degassed for 6 h at 200 °C. The specific surface area of each material was calculated by using the Brunauer – Emmett – Teller (BET) analysis whereas the pore size distribution was obtained according to the Non-Local Density Functional Theory (NLDFT) method. Electrochemical measurements such as cyclic voltammetry (CV) and chronopotentiometry were carried out for the charging-discharging characteristics on a CHI 760C electrochemical workstation, and an impedance spectroscopy measurement was investigated on a ZIVE mp2 electrochemical workstation. The electrochemical measurements of all the samples were carried out in 2 M KOH aqueous electrolyte solution with a standard three-electrode cell. Ag/AgCl and Pt were used as a reference and counter electrode respectively. For the electrochemical measurement, electrode materials were prepared by mixing 90 wt % of MCF-M-T materials (1 mg) and 10 wt % of polyvinylidene fluoride (PVDF) in isopropanol. The slurry was coated onto a nickel foam and the nickel foam was pressed, and dried at 100 °C in the vacuum oven overnight. The CV and chronopotentiometry measurements were carried out in a potential range from -0.6 V to 0.2 V at different scanning rates and current densities. The impedance spectroscopy measurement was carried out using frequency range between 1 MHz and 1 mHz with an amplitude of 0.005 V. In addition, the capacitive performance of MCF-M-150 was further studied using a 2032 two-electrode standard coin cell. The working electrode was prepared by thoroughly mixing with active material of 80 wt%, conductive support of 10 wt% and binder (poly acrylic acid and carboxymethyl cellulose, ratio 5:5) of 10 wt% in ethanol. The slurry was spread onto a cupper sheet and completely dried in the vacuum oven overnight. The coin cell was assembled with 1M Na_2SO_4 as electrolyte and thin polypropylene film as separator. The capacitance was calculated using the equation; $C = 2(I\Delta t/m\Delta V)$, where I is current (A), Δt is the discharging time in (s), ΔV is the range of voltage in (Volts), and m is the weight of the active materials in (g).

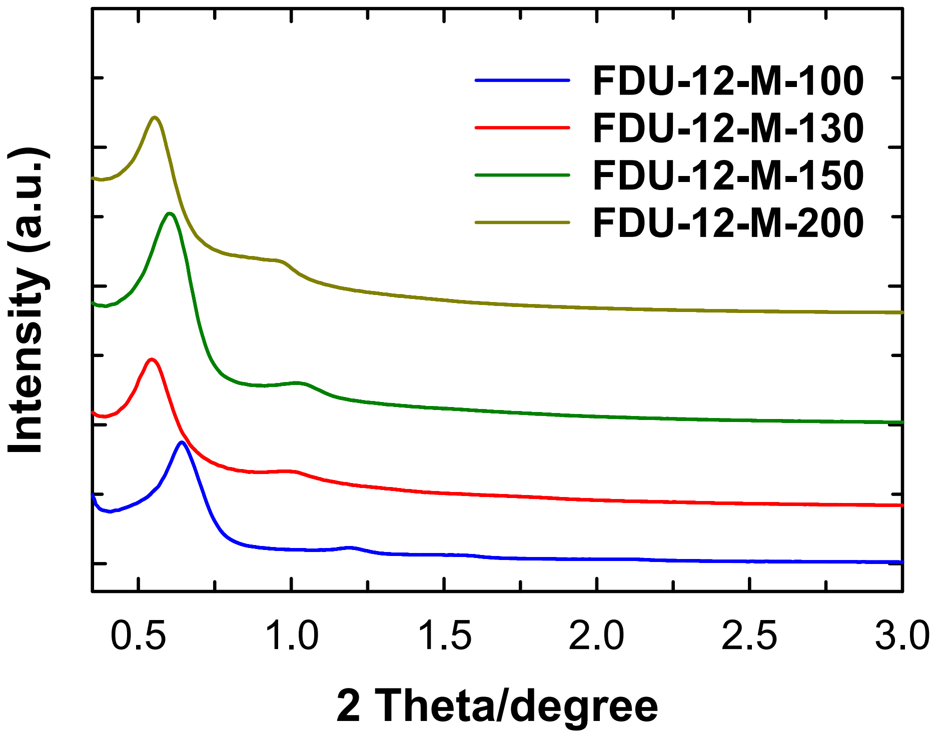

FDU-12-M-100
FDU-12-M-130
FDU-12-M-150
FDU-12-M-200
Intensity (a.u.)
0.5
1.0
1.5
2.0
2.5
3.0
2 Theta/degree

Figure S1: Powder XRD patterns of FDU-12-M-T synthesized by microwave irradiation

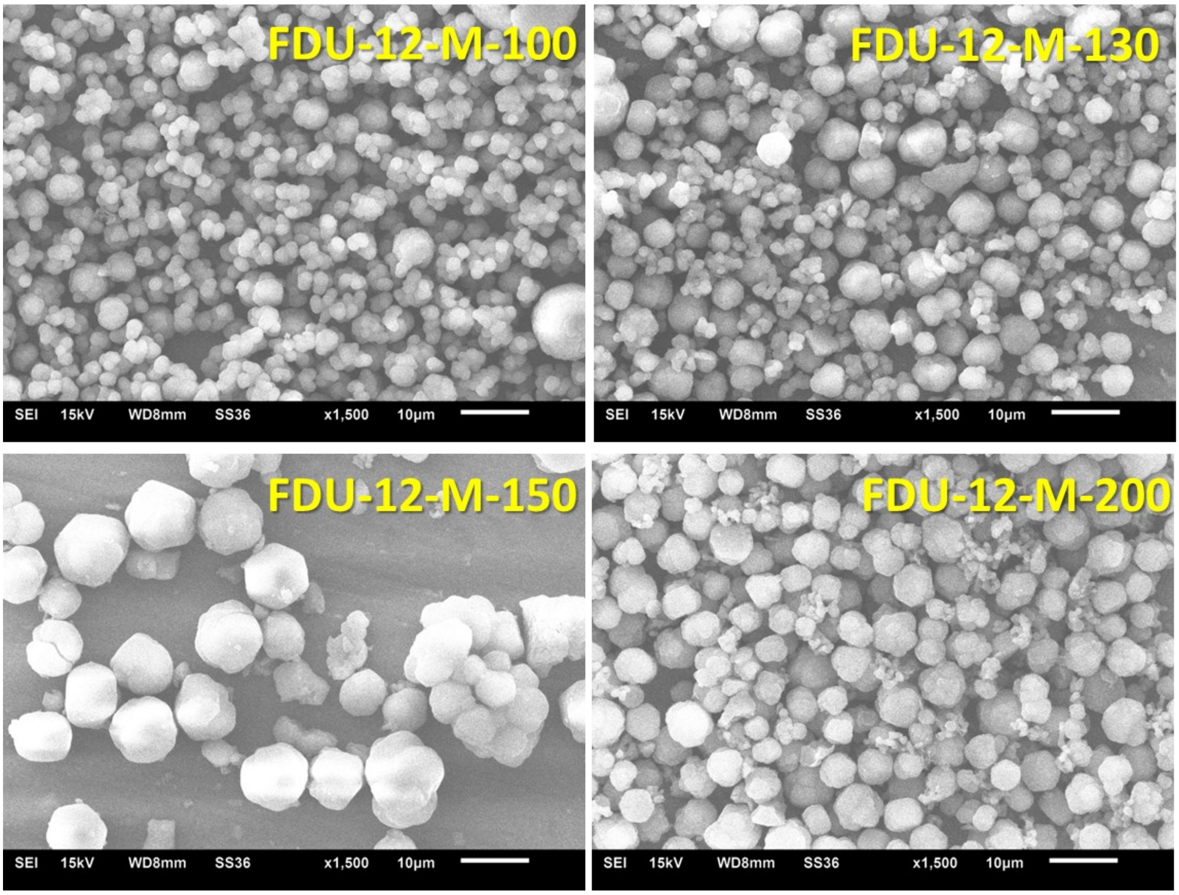

Figure S2: SEM images of FDU-12-M-T samples synthesized by microwave irradiation

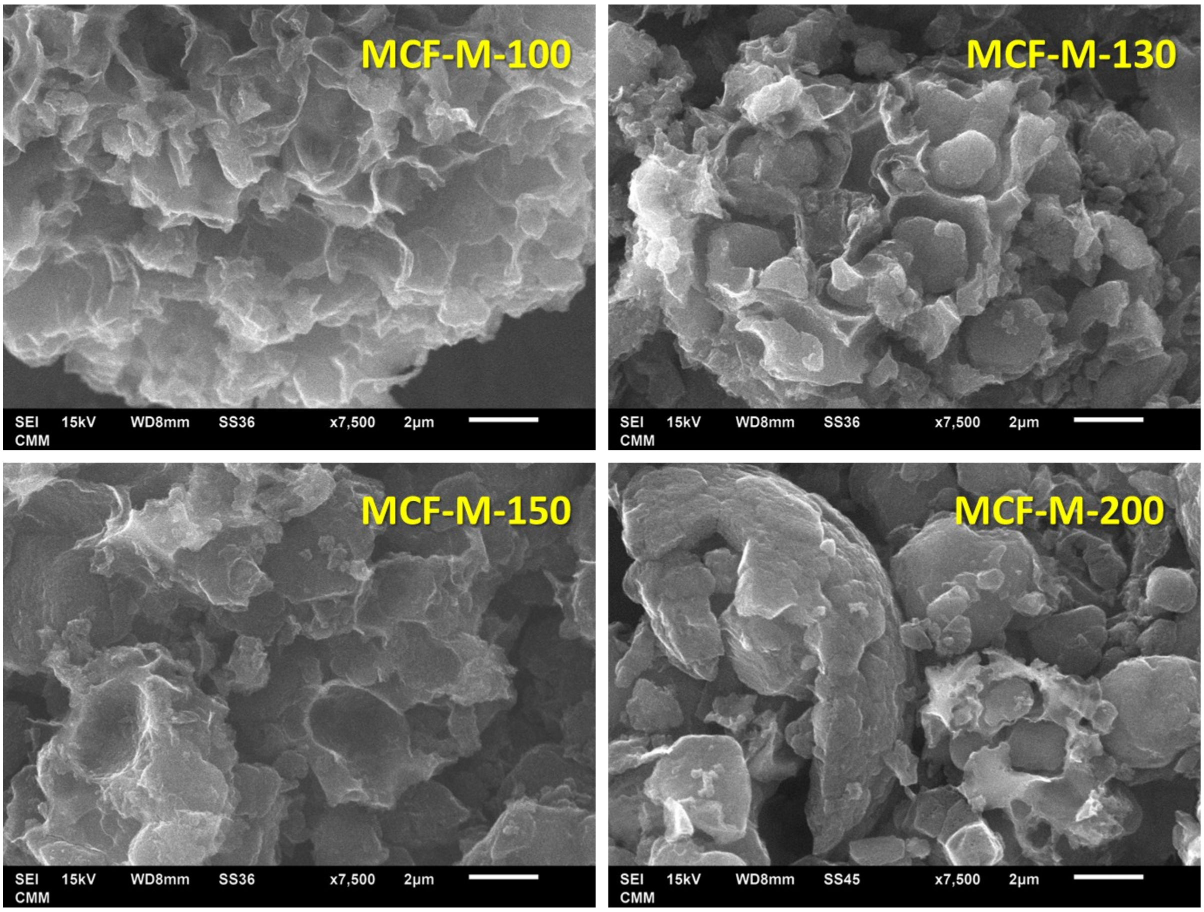

Figure S3: HR-SEM images of MCF-M-T samples synthesized by using FDU-12-M materials by carbonization at 900 ^{o}C for 6h

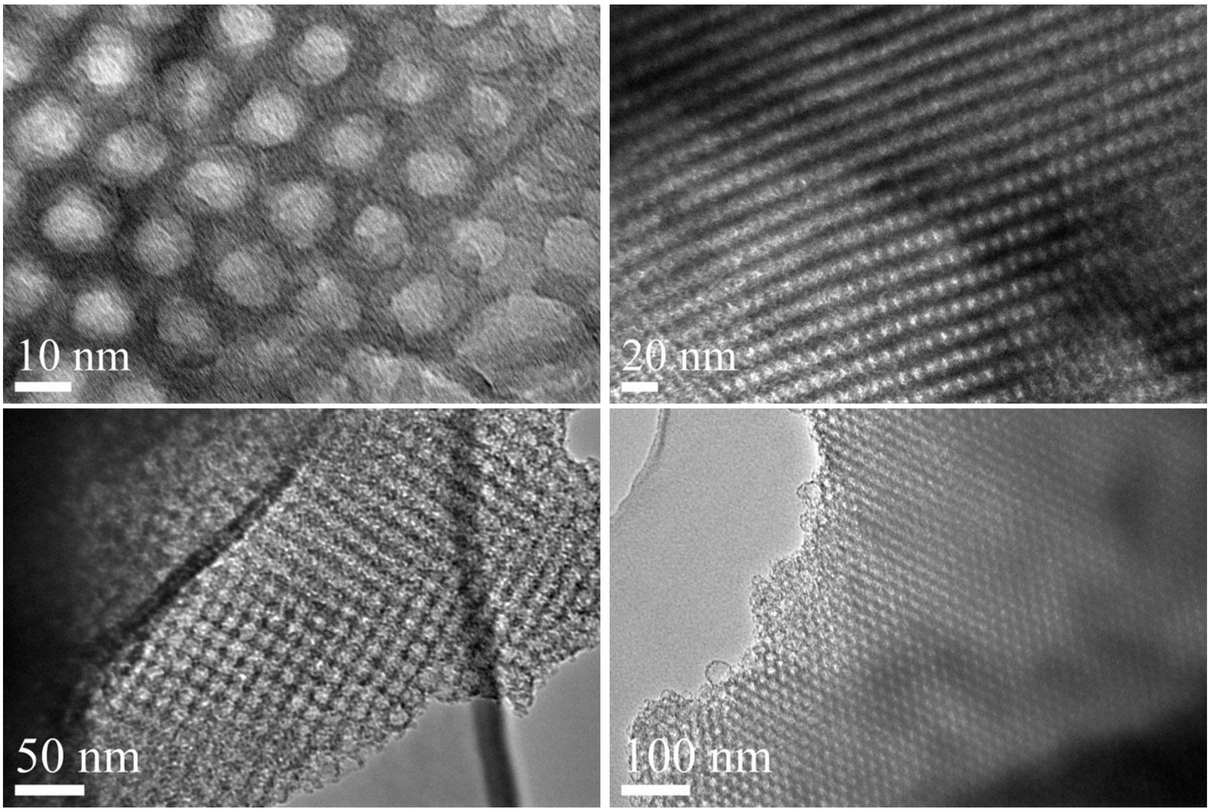

Figure S4: HRTEM images of FDU-12-M-150

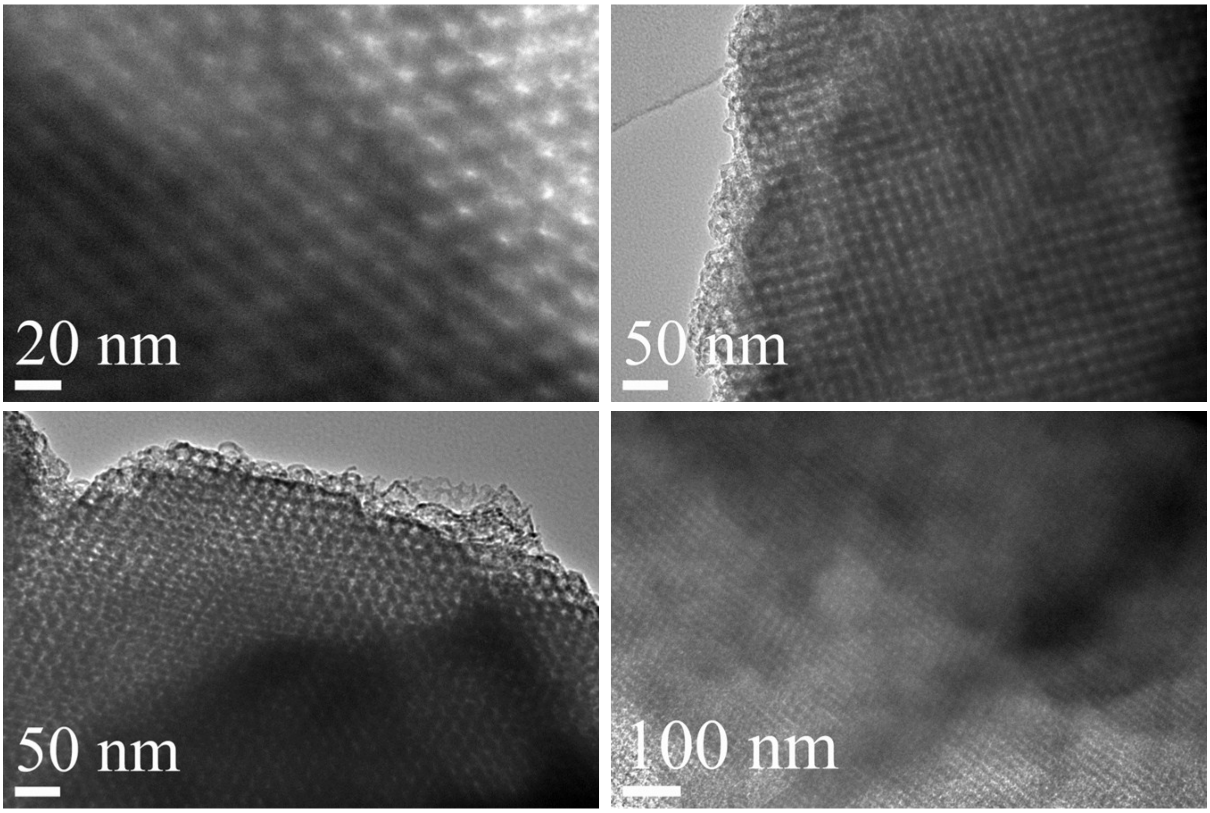

Figure S5: HRTEM images of FDU-12-M-200 sample

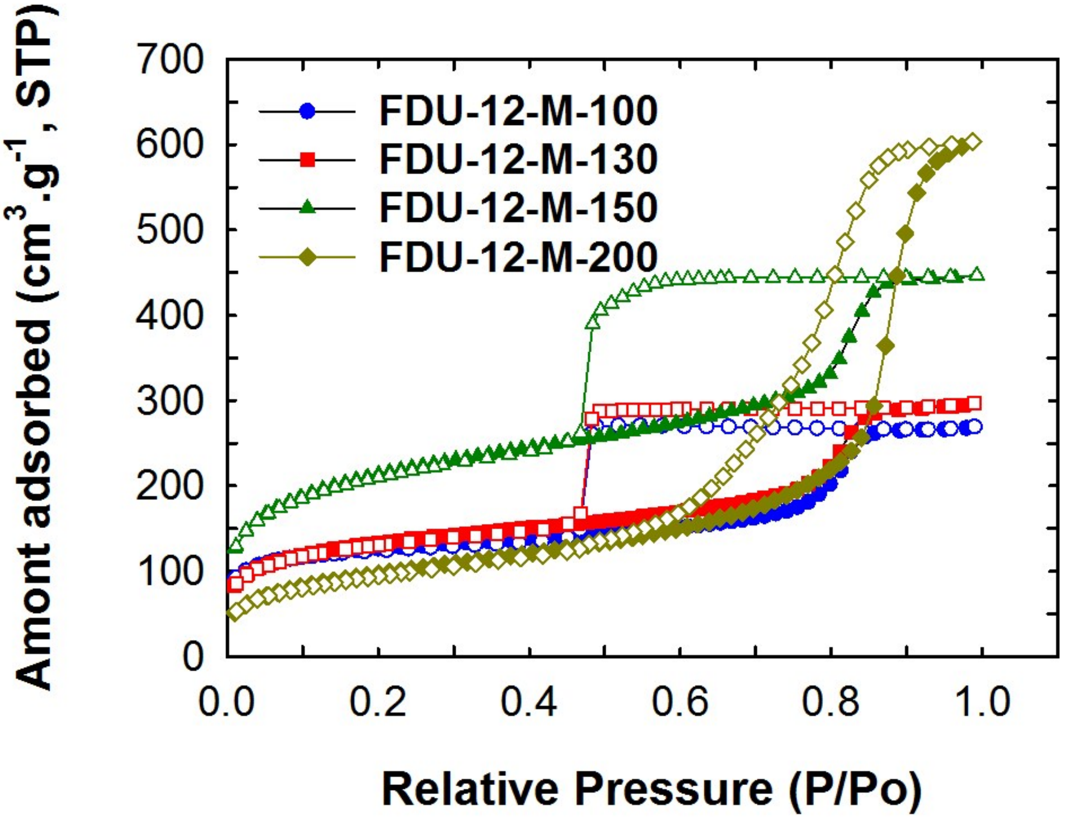

Figure S6: Nitrogen absorption desorption isotherms of FDU-12-M-T

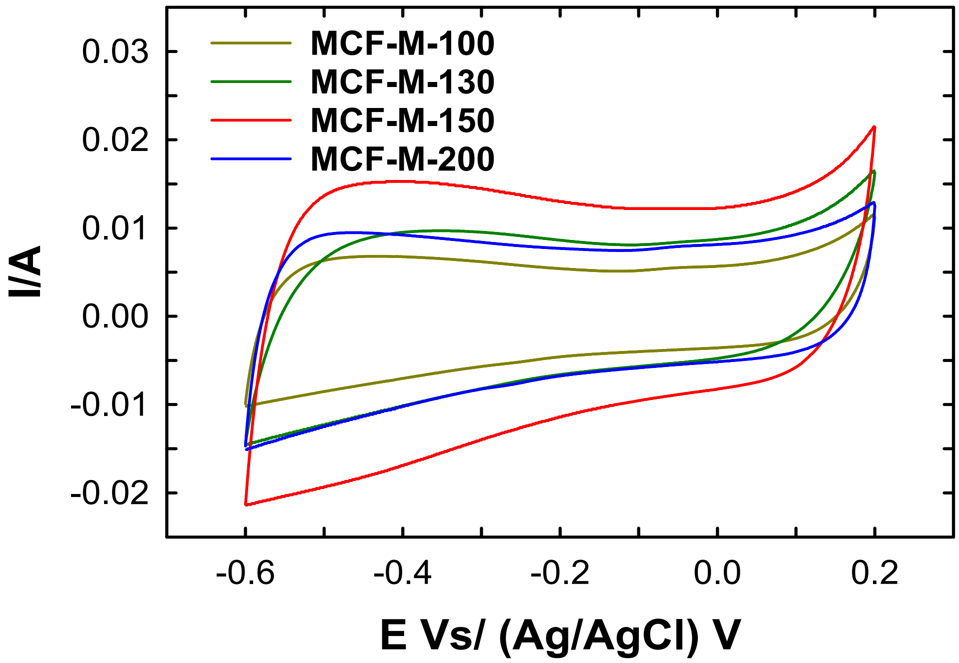

Figure S7: CV profiles of the MCF-M-T samples at scanning rate of 100 mVs^{-1}

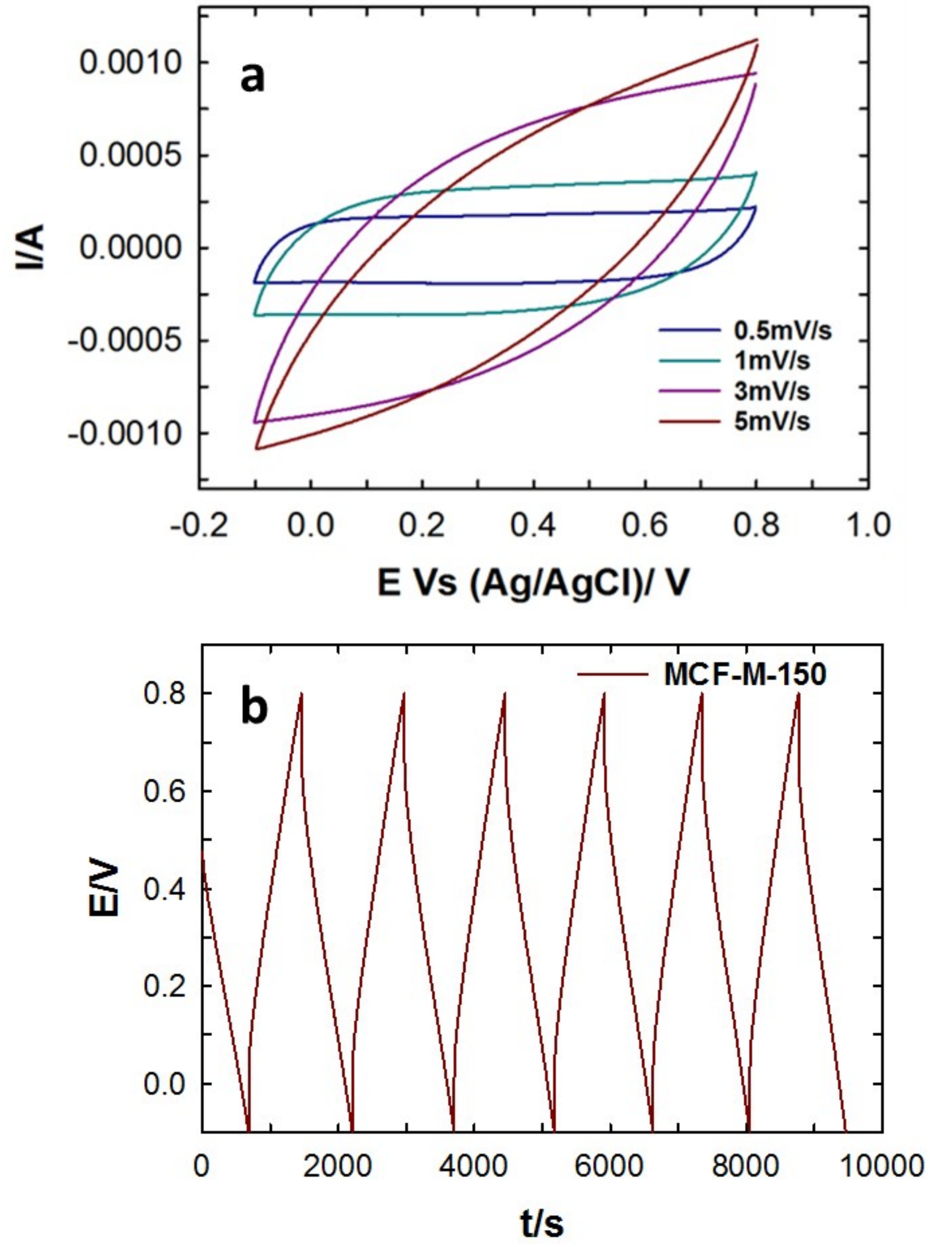

Figure S8: (a) CV profiles of symmetric capacitor of MCF-M-150 at different scanning rate; (b) Charge-discharge profile of symmetric capacitor of MCF-M-150 at current density of 0.15 A g^{-1}.

Table 1S: Comparison of specific capacitance in supercapacitors using carbon based materials showing EDLC behaviour.

Materials	Specific surface area	Specific capacitance	Current density	Electrolyte	Reference
Porous carbon	2339 m^2 g^1	218 F g^{-1}	0.1 A g^{-1}	6M KOH	1
MWCNT	250 m^2 g^{-1}	22F g^{-1}	1 mA cm^{-2}	2M H_2SO_4	2
Activated carbon	747 m^2 g^{-1}	55F g^{-1}	1 mA cm^{-2}	2M H_2SO_4	2
Mesoporous carbon CMK-3	1350 m^2 g^{-1}	186 F g^{-1}	1 mA cm^{-2}	2M H_2SO_4	2
Mesoporous carbon CMK-8	1321 m^2 g^{-1}	176 F g^{-1}	0.625 A g^{-1}	2M KOH	3
MCF-M-150	1251.5 m^2 g^{-1}	315.3 F g^{-1}	1 A g^{-1}	2M KOH	Current work

1 K. Wang, M. Xu, X. Wang, Z. Gu, Q. H. Fan, W. Gibbons, J. Croat, RSC Adv., 2017, **7**, 8236–8240

2 D. S. Dhawale, M. R. Benzigar, M. A. Wahab, C. Anand, S. Varghese, V. V. Balasubramanian, S. S. Aldeyab, K. Ariga, A. Vinu, Electrochim. Acta, 2012, **77**, 256-261

3 J. Lang, X. Yan, X. Y. Yuan, J. Yang, Q.J. Xue, Journal of Power Sources, 2011, **196**, 10472–10478
